# Liver parenchymal sparing surgery for locally advanced gallbladder cancer with extracapsular lymph node invasion

**DOI:** 10.1186/1477-7819-12-183

**Published:** 2014-06-10

**Authors:** Masato Narita, Ryo Matsusue, Hiroaki Hata, Takashi Yamaguchi, Tetsushi Otani, Iwao Ikai

**Affiliations:** 1 Department of Surgery, National Hospital Organization, Kyoto Medical Center, 1-1 Mukaihata-cho, Fukakusa, Fushimi-ku, Kyoto 612-8555, Japan

**Keywords:** Chemotherapy, Extended cholecystectomy, Hepatectomy, Hepatic artery, Liver resection, Morbidity

## Abstract

A complete R0 resection is the standard treatment in patients with gallbladder cancer and the only potentially definitive curative therapy. Major hepatectomy, including right or extended right hepatectomy with extrahepatic bile duct resection, would be an option in patients with locally advanced gallbladder cancer, while morbidity and mortality rate are still high. Herein, we report a case of a locally advanced gallbladder cancer invading the right hepatic artery (RHA), common hepatic duct, and transverse colon. This patient was successfully treated with parenchymal sparing surgery without major hepatectomy and achieved R0 resection by means of extended cholecystectomy combined with resection of the transverse colon, extrahepatic bile duct, and RHA. Intrahepatic arterial flow was preserved without reconstruction of the RHA, and the postoperative course was favorable. Liver parenchymal sparing surgery might be an alternative procedure in patients with gallbladder cancer, to minimize the risk of severe morbidity, if R0 resection is possible.

## Background

Complete surgical resection is the only potentially curative treatment of gallbladder cancer, since the role of chemotherapy and radiation therapy in the management of gallbladder cancer remains undefined [1]. The surgical treatment of locally advanced gallbladder cancer with hepatic arterial invasion is challenging, since surgeons should perform the operation while taking into account both radicality (that is, resection to achieve a R0 resection) and safety (that is, avoidance of fatal morbidity, such as postoperative liver failure). Major hepatectomy, including right hepatectomy or extended right hepatectomy accompanied by extrahepatic bile duct resection, with or without vascular resection or reconstruction, would be an option in these patients, but might be associated with a significant morbidity and mortality even in high volume hepatobiliary centers [2,3].

Therefore, liver parenchymal sparing surgery would be an alternative operative procedure for patients with locally advanced gallbladder cancer accompanied by vascular invasion.

Herein, we report liver parenchymal sparing surgery in a patient with locally advanced gallbladder cancer suspected of extracapsular invasion of the lymph node extended to the right hepatic artery (RHA) and the common hepatic duct. The clinical progress of this patient is reviewed, and the feasibility and rationale of this procedure are discussed in light of the current literature.

## Case presentation

**Figure 1:**
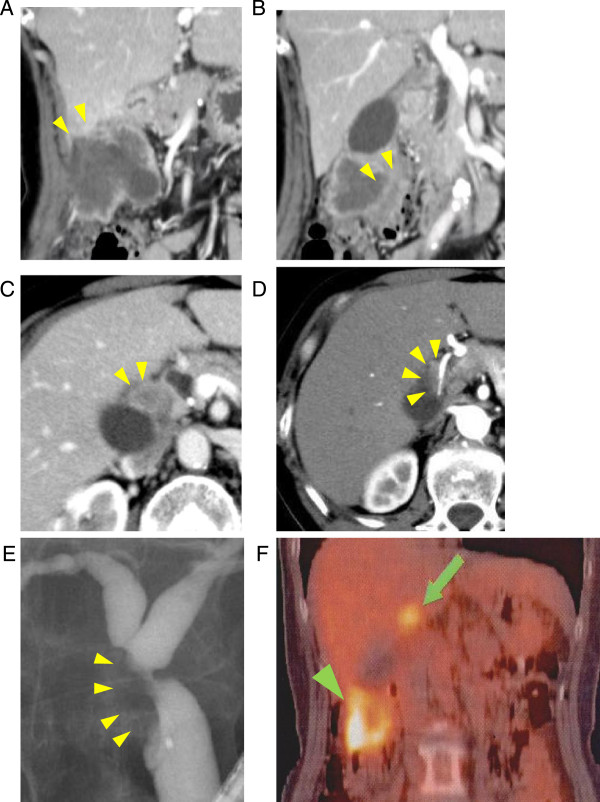
**Preoperative imaging studies. (A-D)** Contrast-enhanced computed tomography (CT). Arrowheads indicate gallbladder tumor with a peripheral wall enhancement extending to the liver parenchyma adjacent to **(A)** the gallbladder and **(B)** the transverse colon. **(C)** Arrowheads indicate an enlarged lymph node with a diameter of 23 mm, which was detected in the area of the cystic duct. **(D)** Arrowheads indicate the right hepatic artery (RHA), which runs close to an enlarged lymph node, suggesting that extracapsular invasion extended to the RHA.** (E)** Endoscopic retrograde cholangiopancreatography: arrowheads indicate a stricture at the level of the junction of the cystic duct and the common hepatic duct. **(F)** Fluorodeoxyglucose positron emission tomography. The arrowhead and arrow indicate high fluorodeoxyglucose uptake in the gallbladder tumor and cystic duct lymph node, respectively.

**Figure 2:**
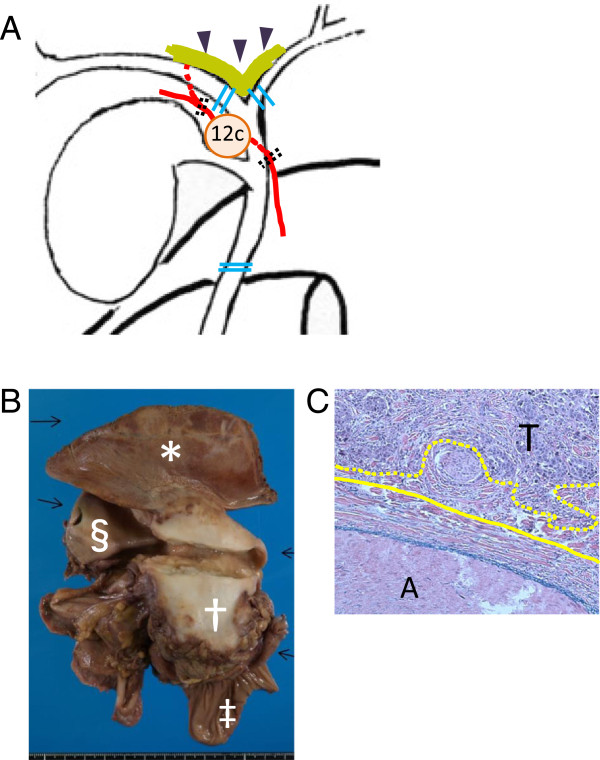
**Schematic illustration of intraoperative finding and pathological examination. (A)** 12c indicates the enlarged lymph node in the area of the cystic duct, which is suspected to invade the RHA and common hepatic duct. Double dotted lines indicate where the RHA is divided. Double lines indicate where the bile ducts are divided. The thick line indicated by arrowheads indicates the preserved hepatic plate, which includes the interlobar hepatic artery running through the Glissonian sheath around the confluence of the hepatic ducts. **(B)** Gross appearance of resected specimens: *, liver parenchyma; †, gallbladder; ‡, transverse colon; §, common hepatic duct. **(C)** Microscopic image of RHA and adjacent tissue (x200). Solid line indicates adventitia of the RHA. Dotted line indicates extracapsular invasion front of gallbladder cancer. A, artery; T, tumor.

**Figure 3:**
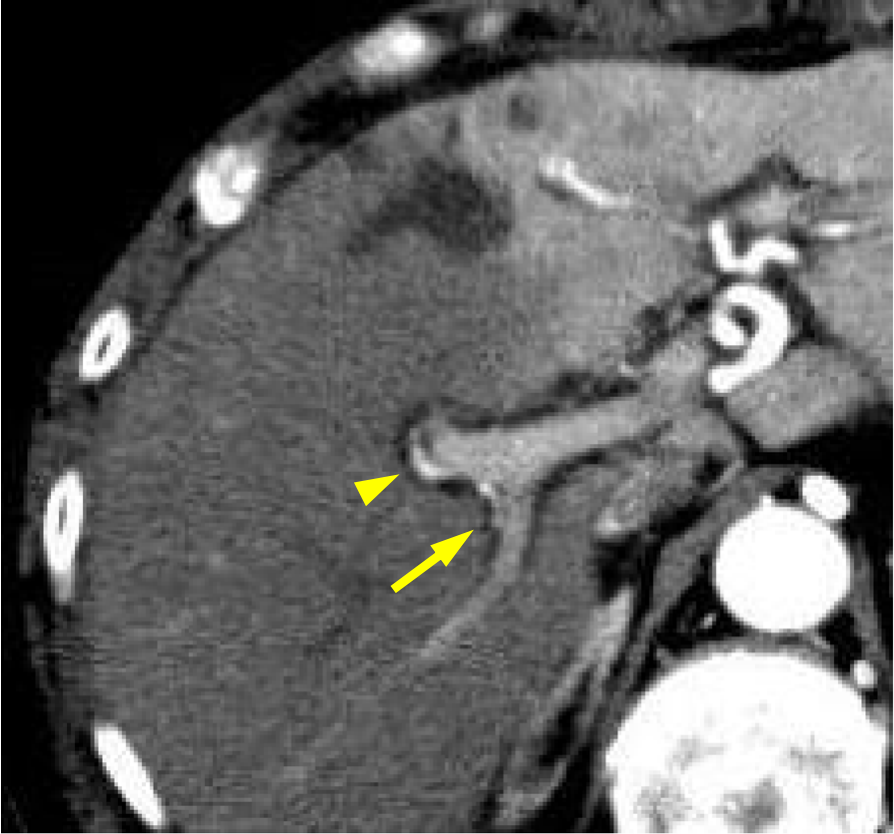
**Postoperative imaging studies using contrast-enhanced CT at (A) postoperative day 1 and (B) 5 months after operation. (A)** Arrowhead and arrow indicate anterior and posterior sectorial branch of the right hepatic artery, respectively.

A 55-year-old woman presented with a painful and large palpable mass at the right upper quadrant of the abdomen. She reported that she had had this for 2 months prior to admission. This patient had no previous history of similar problems or any prior medical or surgical conditions. On physical examination, a right upper quadrant mass with tenderness was palpable. The laboratory examination revealed that the results of liver and renal function tests, urinary analysis, and complete blood count tests were within the normal ranges. Tumor marker levels, carcinoembryonic antigen (CEA:11.8 ng/ml) and carbohydrate antigen 19-9 (CA19-9:1521 U/ml), were elevated. Contrast-enhanced computed tomography (CT) showed a tumor in the fundus and body of the gallbladder, 6 × 4 cm in size, with a peripheral wall enhancement (Figure 1A,B). This tumor extended to the liver (Figure 1A) and transverse colon (Figure 1B). An enlarged lymph node with a diameter of 23 mm was detected in the area of the cystic duct (Figure 1C). The RHA from the proper hepatic artery runs close to this lymph node (Figure 1D), suggesting extracapsular invasion into the RHA. The estimated volume ratio of the left hepatic lobe to the whole liver was 0.3. Endoscopic retrograde cholangiography showed a stricture of the common hepatic duct and deficit of the cystic duct (Figure 1E). Fluorodeoxyglucose positron emission tomography showed high fluorodeoxyglucose uptake (accumulation) in the gallbladder tumor and cystic duct lymph node (Figure 1F), while there was no evidence of distant metastasis. Taken together with these examinations, this patient was diagnosed with locally advanced gallbladder cancer extending to the liver parenchyma and the transverse colon with lymph node metastasis in the area of the cystic duct, invading the RHA and common hepatic duct. We planned surgical intervention for curative intent since there was no evidence of distant metastasis.At laparotomy, neither peritoneal dissemination nor distant metastasis was detected. The transverse colon invaded by the tumor was partially resected. The lower common bile duct was dissected after its ligation as low as possible in the pancreas. The enlarged lymph node was involved in the RHA and tightly adhered to the common hepatic duct. This finding strongly indicated extracapsular invasion of the lymph node, extending to the RHA and the common hepatic duct. Intraoperative Doppler ultrasonography showed sufficient right intrahepatic arterial flow despite the clamp of the RHA. We speculated that the right intrahepatic arterial flow would be preserved by the interlobar hepatic artery at the hepatic hilum, perfused from the left hepatic artery. The patient underwent extended cholecystectomy with a 2-cm wedge resection of the liver parenchyma as a safety margin combined with regional lymph node dissection. The RHA was dissected at both the distal and proximal side, as much as possible, and divided without reconstruction. The right and left hepatic ducts were carefully dissected and divided with a preservation of the communication across the hilar plate to avoid injury of the interlobar hepatic artery (Figure 2A). After this procedure, a specimen of the tumor substance was removed. The right intrahepatic arterial flow was detected by Doppler ultrasonography. Separate orifices of the right and left hepatic ducts were joined to form a single orifice and biliary reconstruction was performed using a Roux-en-Y hepaticojejunostomy. The duration of operation was 485 minutes and the amount of blood loss was 380 g without blood transfusion.Pathological examination revealed moderately differentiated adenocarcinoma situated mainly in the body and fundus of gallbladder, invading the transverse colon, liver parenchyma, and lymph node in the area of the cystic duct (Figure 2B). The neck of the gallbladder was intact. There was no evidence of lymph node involvement in the hepatoduodenal ligament, behind the pancreatic head or in the common hepatic artery region. Extracapsular invasion of the lymph node extended to the common hepatic duct and periadventitial tissue of the RHA, while there was no evidence of invasion of the adventitia of the RHA (Figure 2C). R0 resection was achieved. According to the International Union Against Cancer (UICC) classification system, pathological staging was pT3N1(1/16)M0, Stage IIB.At postoperative day 1, the bilirubin concentration in the drain fluid was 17.3 mg/dl, indicating leakage of the bilioenteric anastomosis. Contrast-enhanced CT was performed on the same day and revealed that there was no evidence of intra-abdominal fluid collection. Intrahepatic arterial flow was preserved in the right liver lobe, although parenchymal enhancement was delayed (Figure 3). Postoperative changes in serum biochemistry included a rapid increase of transaminase (aspartate transaminase, 830 U/l; alanine transaminase 495 U/l), lactate dehydrogenase (949 u/l), and total bilirubin (3.1 mg/dl) on postoperative day 1, while they decreased gradually after postoperative day 2. The leakage of the bilioenteric anastomosis was treated conservatively with continuous drainage, since the patient’s general condition was stable. This patient was discharged at postoperative day 45 and the drainage tube was removed at postoperative day 60.

Computed tomography was performed at 5 months after operation and indicated liver metastasis within the left lateral liver. There was no evidence of local recurrence at the cut surface of the liver parenchyma or around the hepaticojejunostomy. Subsequently, the patient was given chemotherapy using combination of gemcitabine and cisplatin. The patient tolerated the chemotherapy well and long-term disease stability was achieved. After 13 cycles of chemotherapy, second-line chemotherapy using an oral 5-fluorouracil derivative (S-1) was begun, taking into account of disease progression. Computed tomography performed after four cycles of chemotherapy revealed progression of liver metastasis. The patient died 31 months after surgery.

## Discussion

In general, a major hepatectomy including extended right hepatectomy or right hepatectomy would be performed, in which gallbladder cancer might involve either the right Glissonian sheath, the hepatic hilum, the RHA, or the portal vein [4]. The present case was one of locally advanced gallbladder cancer, with suspected extracapsular invasion of the lymph node extended to the RHA and the common hepatic duct, as determined by preoperative imaging studies. This patient underwent an alternative surgical procedure, extended cholecystectomy combined with resection of the RHA, for the following three reasons: (1) preoperative imaging studies indicated that RHA invasion was not due to direct invasion of the gallbladder cancer but to extracapsular invasion of the lymph node, suggesting that major hepatectomy would not be necessary to achieve R0 resection; (2) right intrahepatic arterial flow was preserved even at the clamp of the RHA; (3) the left hepatic lobe was relatively small and simultaneous resection of the transverse colon was required.

Miyazaki *et al.*[5] reported that in the case of biliary tract carcinoma involving a unilateral hepatic artery, such as the RHA, arterial reconstruction is not always requisite when the communication across bilateral hepatic bile ducts wall is preserved, because the interlobar hepatic artery runs through the Glissonian sheath around the confluence of the hepatic ducts. In this case, the hepatic duct was carefully resected with a preservation of the communication across the bilateral hepatic bile duct wall, to avoid injury of the interlobar hepatic artery. As a result, the communication between major lobar arteries and intrahepatic arterial flow were preserved, as shown in the CT scan from postoperative day 1, although parenchymal enhancement was delayed. This finding suggested that the flow of blood in the RHA decreased. The deprivation of hepatic arterial flow in patients with a bilioenteric anastomosis has been reported as usually resulting in leakage of bilioenteric anastomosis and extensive liver infarction of the dearterialized region [6,7]. In this case, leakage of bilioenteric anastomosis developed in the early postoperative course but was cured conservatively without major problems. This complication might be because the biliary tree is vulnerable to ischemia resulting from a dearterialization [6]. Therefore, surgeons should be aware of an increased risk of biliary fistula in this procedure.

The extent of hepatic resection required in patients with locally advanced gallbladder cancer is still controversial. Major hepatectomy might be an option in such cases, for two reasons: (1) ensuring an adequate tumor-free margin, and (2) to remove microscopic tumor foci potentially present within the right half of the liver, particularly in the vicinity of the gallbladder bed [8]. However, liver metastasis in both liver lobes is the most frequent type of hepatic recurrence [9]. In this case, cancer relapse developed only within the left lateral lobe. Therefore, prophylactic resection of the liver parenchyma to prevent liver metastasis might be insufficient to achieve long-term survival in patients with advanced gallbladder cancer. The postoperative long-term outcome in these patients was poor even in patients undergoing R0 resection. An immediate and sufficient cause of adjuvant chemotherapy might be a key to improve the prognosis in these patients. From this point of view, parenchymal sparing surgery with R0 resection might be superior to major hepatectomy in terms of minimizing the risk of severe morbidity, and achievement of chemo-tolerance by preservation of sufficient liver function. In fact, a tolerance of chemotherapy in our case was achieved, resulting in long-term disease stability.

Reddy *et al.*[10] reported in a multi-institutional study that simultaneous colorectal and major hepatic resection was associated with a high incidence of severe morbidity, although simultaneous colorectal and minor hepatic resection could be performed safely, as could staged hepatic resection. To minimize the risk of severe morbidity, we performed wedge resection of the liver with adequate surgical margin combined with partial colectomy to succeed in achieving R0 resection.

## Conclusions

In conclusion, we safely performed parenchymal sparing surgery without sacrificing oncological radicality in a patient with locally advanced gallbladder cancer suspected of extracapsular invasion extended to the RHA and the common hepatic duct. Bile leakage developed early in the postoperative course, but was cured conservatively without major problems. Although cancer relapsed within the left liver lobe 5 months after the operation, this patient survived about two years after disease recurrence. The prognosis for advanced gallbladder cancer is quite poor and R0 resection is an essential factor for long-term survival. Aggressive surgical intervention is usually required in such cases, while if R0 resection is possible, liver parenchymal sparing surgery might be useful, depending on the circumstances (that is, patients with a small left hepatic lobe, or requiring simultaneous resection of digestive tract), to minimize the risk of severe morbidity and for tolerance of chemotherapy.

## Consent

Written informed consent was obtained from the patient’s next of kin for publication of this case report and any accompanying images. A copy of the written consent is available for review by the editor-in-chief of this journal.

## Abbreviations

CT: computed tomography; RHA: right hepatic artery; UICC: International Union Against Cancer.

## Competing interests

The authors declare that they have no competing interests.

## Authors’ contributions

Study concept and design: MN and II. Acquisition of data: MN and II. Analysis and interpretation of data: MN, RM, HH, TY, TO, and II. Drafting of the manuscript: MN and II. Critical revision of the manuscript for important intellectual content: RM, HH, TY, and TO. Study supervision: II. All authors read and approved the final manuscript.
